# ISQ calculation evaluation of in vitro laser scanning vibrometry-captured resonance frequency

**DOI:** 10.1186/s40729-017-0105-3

**Published:** 2017-10-12

**Authors:** Stijn Debruyne, Nicolas Grognard, Gino Verleye, Korneel Van Massenhove, Dimitrios Mavreas, Bart Vande Vannet

**Affiliations:** 1 Department of Mechanics, Research Group Propolis, School of Engeneering Sciences, Katholieke Hoge School Brugge-Oostende, Ostend, Belgium; 2 Kliniek Royal, Koningstraat 41, 8400 Ostend, Belgium; 3 CHIR-Unit Dentistry–ORHE, Department of Orthodontics, Faculty of Medicine and Pharmacy, Vrije Universiteit Brussel, Laarbeeklaan 103, 1090 Brussels, Belgium; 4 Department of Communication Sciences, Ghent University, Korte Meer 7-9-11, 9000 Ghent, Belgium; 5 Katholieke Hoge School Brugge-Oostende, Ostend, Belgium

**Keywords:** Implant stability, RFA, ISQ, Osstell Mentor, Osstell IDx, Laser Doppler vibrometry, Straumann tissue level implants, Ankylos implants

## Abstract

**Background:**

Implant stability testing at various stages of implant therapy by means of resonance frequency analysis is extensively used. The overall measurement outcome is a function of the resulting stiffness of three entities: surrounding bone, bone-implant complex, and implant-Smartpeg complex. The influence of the latter on the overall measurement results is presently unknown. It can be investigated in vitro by use of imbedded implants with mounted Smartpegs. This enables to keep the influence of the two other entities constant and controlled.

The purpose of this study is to verify if a laboratory laser Doppler vibrometry technology-based procedure results in comparable ISQ results after calculation of captured resonance frequency spectra by aid of the Osstell algorithm with direct Osstell IDX device measurements.

**Methods:**

A laboratory procedure was engineered to record frequency spectra of resin-imbedded test implants with mounted Smartpegs, after electromagnetic excitation with the Osstell IDX device and laser Doppler vibrometry response detection. Fast Fourier transformation data processing of resonance frequency data resulted in determination of a maximum resonance frequency values allowing calculation of implant stability quotient (ISQ) values using the Osstell algorithm.

**Results:**

Laboratory-based ISQ values were compared to Osstell IDx device-generated ISQ values for Straumann tissue level, Ankylos, and 3i Certain implant systems. For both systems, a correlation coefficient *r* = 0.99 was found. Furthermore, a clinically rejectable mean difference of 0.09 ISQ units was noted between both datasets.

**Conclusions:**

The proposed laboratory method with the application of the Osstell algorithm for ISQ calculation is appropriate for future studies to in vitro research aspects of resonance frequency analysis implant stability measurements.

## Background

At present, multiple implant stability assessment methodologies are used, both of invasive and non-invasive nature, including percussion test [1], X-ray evaluation [2], cutting resistance during implant insertion (e.g., electronic insertion torque determination) [3], turn-out or reverse torque test [4], Periotest® [5, 6], and resonance frequency analysis (“RFA”), e.g., the Osstell method [7, 8]. The validity of those methods can be evaluated to their sensitivity to detect small changes in stability that are not detectable with clinical and/or radiographical methods. Electronic devices such as insertion torque devices, Periotest, and Osstell Mentor devices are commercially available instruments that allow quantitative implant stability analysis at a level that is not feasible with traditional clinical or radiographical methods [9]. The Osstell device methodology is based on quantitative assessment of (micro) deflection of the implant—by aid as a transducer—in the surrounding jawbone, induced by controlled appliance of electromagnetic excitation. The properties of a transducer (e.g., stiffness and screw properties), the stiffness of the “implant-transducer” complex, the properties and stiffness of “implant-bone” complex, e.g., the effective height of the coronal implant part above the bone crest [7, 8], and the stiffness of the bone itself are measurement influencing factors (Integration Diagnostics Company®, Osstell mathematical explanation, 2009).

In the past, various versions of the Osstell device  have been developed and marketed. The original version consisted of a wired version of the transducer. The transducer consisted of two built-in piezoceramic elements. One piezoceramic element served as the transmittor element, receiving an electrically generated sine wave with varying frequency. The response signal was analyzed by an oscilloscope with the resonance frequency in kilohertz as the outcome unit.

The launch of the Osstell Mentor® in 2004 included the introduction of a less voluminous, much more user-friendly, non-cabled transducer, called Smartpeg. Smartpegs are small aluminum rods with three different parts: a coronal part with an implant system-specific screw fitting into the individual implant, a hexagon part enabling easy tightening/un-tightening, and a magnet serving as the electromagnetic puls captor. The apparatus itself was a compact device with an incorporated microcomputer and electromagnetic signal emitting and receiving tipped probe. Excitation of the implant-mounted Smartpeg is performed by four electromagnetic pulses with different frequencies inducing Smartpeg vibration in mostly two directions perpendicular to each other. The vibration directions correspond to a low and a high resonance frequency. The manufacturer recommends performing at least two measurements, in order to identify these possible different stabilities. Furthermore, in order to suppress electromagnetic environmental noise, the working principle is refined by four times repeated emission of each excitation frequency. In summary, 16 pulses are emitted for each single measurement. The captured outcome of each in fourfold emitted signal is converted by the built-in microcomputer into a frequency spectrum by a “fast Fourier transformation” (FFT) method, ending up to detect among the four calculated spectra the two highest peaks representing the resonance frequencies of the implant. The latter will be used to calculate the so-called implant stability quotients (ISQ) by aid of a mathematical algorithm. ISQ is a unitless number, ranging between 0 and 100. If the difference between the two peaks is less than 3 ISQ units, or in case of only one peak detection, only 1 ISQ value will be displayed by the microcomputer. The 2009 Osstell ISQ version and the present Osstell IDx® version do make use of the same algorithm.

The computed ISQ value is based on the following calculation formulae:

$$ \mathrm{ISQ}=\left(f4\times e\right)+\left(f3\times d\right)+\left(f2\times c\right)+\left(f\times b\right)+a $$

Hereby, *f* denotes the measured maximum resonance frequency (RF). Coefficients *a*, *b*, *c*, *d*, and *e* are property information of Osstell (Osstell AB, Gothenburg, Sweden). The coefficients were provided for internal use under the agreement of no publication. From clinical reports [10–16] listed in Table 1, it can be concluded that ISQ values for one specific implant system, inserted in comparable jawbone regions, differ considerably between outcomes obtained by the original wired Osstell device and the more recent Osstell Mentor device. These findings were confirmed in both a clinical trial with an approximate difference of 9 ISQ units [16] and in vitro on human cadaver jawbone with an approximate difference of 10 ISQ units [17]. This means that the comparison of clinical studies reporting implant stability outcomes generated by different versions of Osstell devices in (systematic) reviews needs caution and correction.

**Table 1:** Published secondary implant stability values for Straumann tissue level RN SLA surfaced implants (Ø = 4.1 mm)

Author and study	Implant position (implant number)	Mean ISQ values at given time-point post-insertion	Type of Osstell device used
Barewal et al. 2003 [10]	UJP (10) LJP (17)	8 W: 58 8 W: 62.5	Wired Osstell transducer
Bisschof et al. 2003 [11]	UJP (54) LJP (36)	12 W: 57.1 12 W: 64.7	Wired Osstell transducer
Huwiler et al., 2006 [12]	UJP + LJP (17)	8 W: 62.8	Wired Osstell transducer
Han et al. 2009 [13]	LJP + LJP (10)	8 W: 75.2 12 W: 78.8	Osstell Mentor
Bornstein et al. 2009 [14]	LJP (56)	7 W: 81.1 12 W: 83.3	Osstell Mentor
Guler et al. 2013 [15]	UJP + LJP (108)	8 W: 71.2	Osstell Mentor

### Purpose

**Fig. 1:**
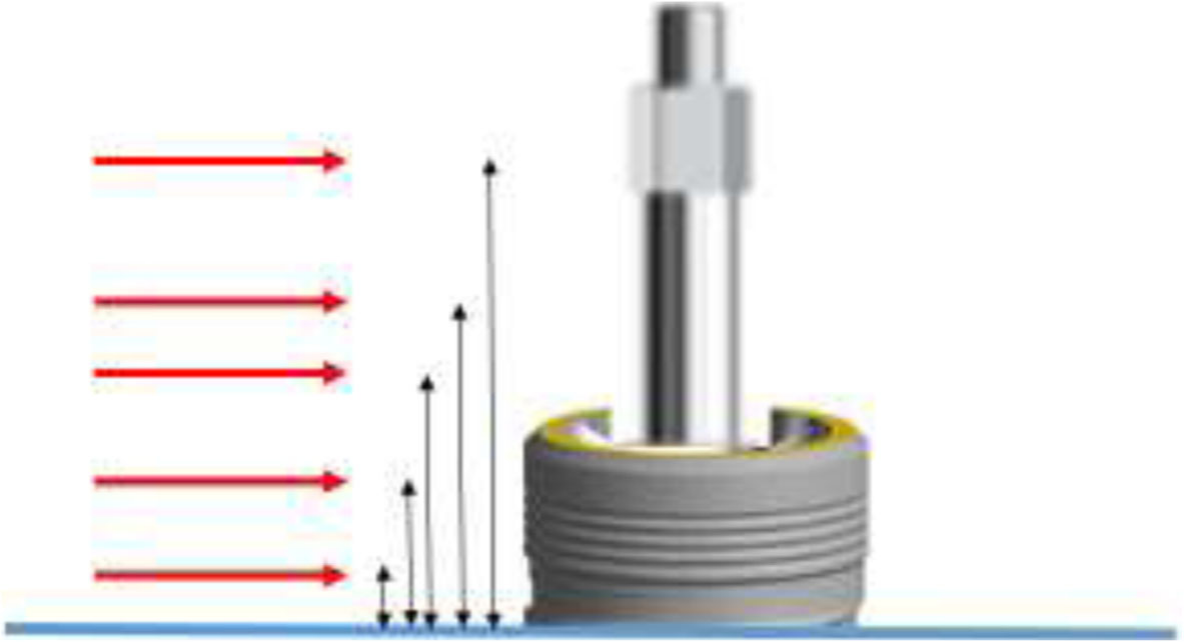
Concept for study of deflection and stiffness aspects of implant-Smartpeg complex by laser Doppler vibrometry. Intentional partial imbedding of implants allows to detect both the deflection of implant and Smartpeg separately at different vertical levels by changing the position of the laser beam

The purpose of this in vitro study was to develop a laboratory method, intended for future research of aspects of implant-Smartpeg complex stiffness and its possible influence on the overall RFA-based implant stability determination. For this, a combination of laser Doppler vibrometry for measurement and signal processing by aid of fast Fourier transformation analysis was used. Laser Doppler vibrometry technology permits to determine both the resonance frequency and deflection behavior of a mounted Smartpeg. The latter can be of interest since different implant types possess different prosthetic connections that suit different types of Smartpegs. In vitro research enables to control and simulate in a standardized way the stiffness of the surrounding bone and the stiffness of the implant-bone complex by imbedding implants in self-curing resin. Complete imbedding of the implant to the most coronal level simulates a normal clinical situation of total osseointegration. Incomplete imbedding allows to both measure the deflection mode of the Smartpeg and the implant itself when different vertical points are used to execute the measurement (Fig. 1).

Laser Doppler vibrometry possesses a working principle based on the so-called Doppler effect and allows non-contact quantitative measurement of vibration (https://en.wikpedia.org/wiki/Laser_scanning_vibrometry, 2017). The Doppler effect itself finds its origin when a light beam is backscattered on a vibrating surface and experiences a change in wave phase (https://en.wikipedia.org/wiki/Doppler_effect, 2017). The backscattered laser beam is captured by the laser scanning vibrometer, and the phase change will be the function of the magnitude of the vibration of the Smartpeg. The response signal is processed and points to maximum detected resonance frequency that will be used to compute the ISQ value by means of the algorithm used by Osstell methodology. The calculated ISQ value is compared to ISQ values generated by the newest version of the Osstell device, Osstell IDx®, using a laboratory setup that enables to capture and measure, by means of laser Doppler vibrometry, the generated electromagnetic excitation of an implant-mounted Smartpeg® transducer, evoked by a the Osstell IDx device*.* The coefficients implemented in the formulae were confidently supplied under the agreement that publication will not be done. This computed ISQ value will be compared to the ISQ value, obtained by the Osstell IDx device in the same laboratory setup.

## Methods

### Test implants

Test implants originating from various manufacturers were investigated. Straumann sandblasted, large-grit, acid-etched (SLA)® tissue level standard implants (Straumann AG, Basel, Switzerland) with the following diameter: length configurations were 3.3–12 mm (RN connection), 3.3–4.1 mm (RN connection), and 4.8–8 mm (WN connection), Ankylos Cell Plus® surfaced B-implant types (Dentsply Implants, Mannheim, Germany) with the following diameter: length configurations used were 4.5–8 mm and 4.5–9.5 mm, and Biomet 3i Full Osseotite® Tapered Certain implants (Biomet 3i, Barcelona, Spain) with a 4-mm diameter/13-mm length were investigated.

### Preparatory procedures

Test implants were imbedded using Duromod B® dual component polyurethane resin (Dumont Instruments, Brussels, Belgium) in a silicon mould with a bar-shaped recipient (approximal dimensions (length × width × height): 16 cm × 2.5 cm × 3 cm)). Per bar, five implants were imbedded using system-specific implant mounts allowing correct vertical positioning. After resin polymerization, all implants were given an identification number in order to allow transfer of the measurement outcomes to the datafile.

### Smartpeg connection

A fresh implant system-specific Smartpeg® (Integration Diagnostics AB, Säveden, Sweden) transducer was connected to each implant using a manual torque controlling device set at 8 Ncm (Tochnichi, Ota-Ku, Tokyo, Japan). For Straumann implants, Smartpeg type # 04 and, for Ankylos implants, Smartpeg type # 16 were used. For 3i Tapered Certain implants, Smartpeg type # 15 was used.

### Laboratory setup for indirect measurements

**Fig. 2:**
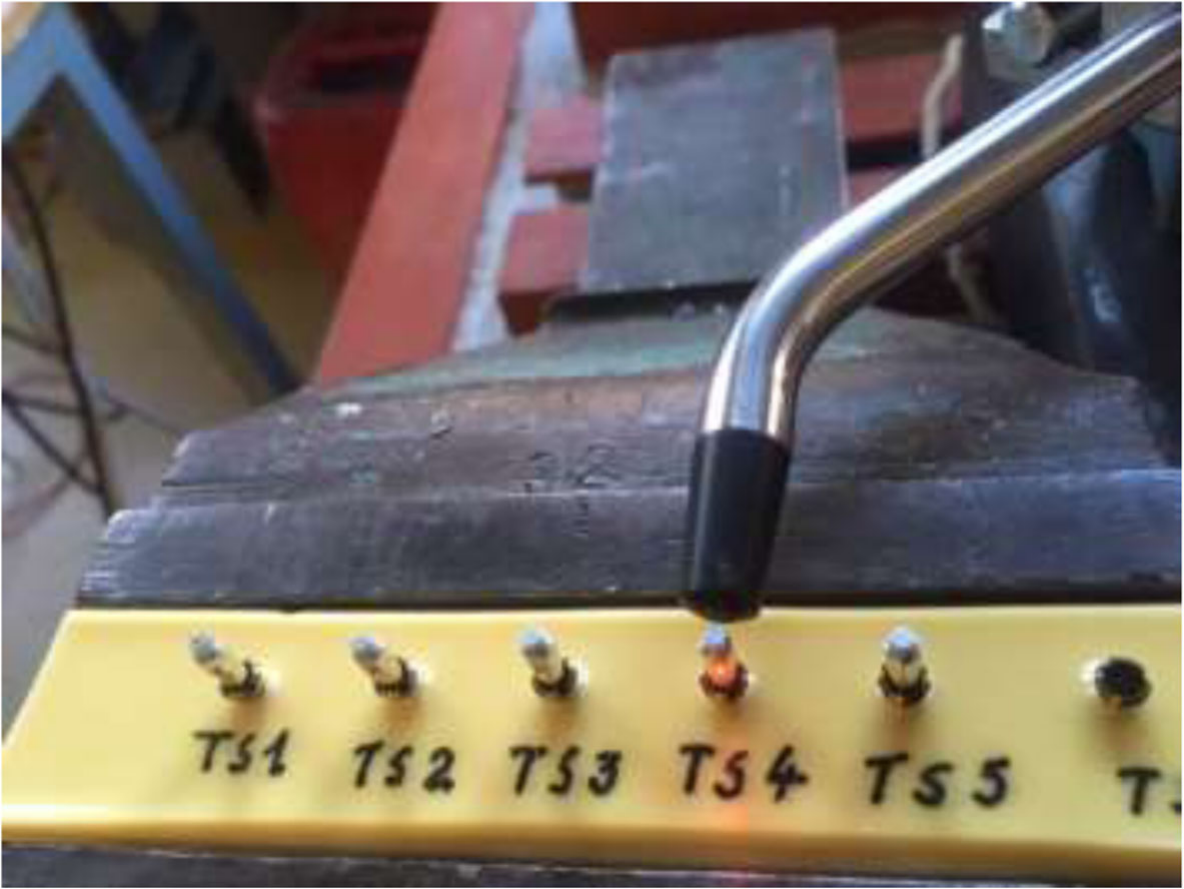
Clamped Osstell probe orientated towards a Smartpeg mounted on a test implant. Note the red laser beam dot on the flat surface of the Smartpeg hexagon part

Smartpeg excitation was performed by using the Osstell IDx device. The cabled probe of the Osstell IDx was positioned towards the most coronal part of the Smartpeg by the aid of a stand (Mitutoyo 70105N, Mitutoyo, Santo Amaro, Brazil) (Fig. 2). The measured ISQ value was noted and input in the datafile.

The speed of vibration, *v*(*t*), of an excited Smartpeg was measured by means of a portable laser vibrometer (laser class 2) (Polytec PDV 100, Polytec, Irvine, CA, USA), generating a focusable laser beam (*λ* = 640 nm), mounted on a tri-pod with a three-way tilting head (Manfrotto, Cassolo, Italy) allowing for easy and precise laser beam orientation in *X*, *Y*, and *Z* directions. The measurement range was set at 20 mm/s with a sensitivity of 5 mm/s. The generated laser beam was orientated towards a flat surface of the hexed part of an implant-mounted Smartpeg. Correct positioning of the laser beam orientation was by visual inspection of laser dot position on a flat surface of the Smartpeg hexagon and by using the reflection index on the laser scanning vibrometer device.

### Laboratory setup for direct ISQ determination

The Smartpeg excitation mode was exactly performed as described above. Notation of the maximum resonance frequency for indirect measurements is followed by notation of direct ISQ value on the display of Osstell IDx device. Positioning of the probe was not changed during indirect and direct recordings for a given test implant.

### Measurements and calculations

Each resin block contained five identical implants with attached Smartpegs of a given implant type with a specific diameter and length configuration. Correct positioning of the Osstell probe towards the Smartpeg magnet part was confirmed by the auditory signal generated by the Osstell device. Correct positioning of the vibrometer laser beam was checked by the visual reflection indicator on the vibrometer display.

The output signal of the laser Doppler vibrometer was linked through an ADC/frontend interface (3160-A-4/2 (Bruëll & Kjaër, Nærum, Denmark) to a laptop with signal processing software (Bruëll & Kjaër Pulse Labshop, Bruëll & Kjaër Nærum, Denmark) to convert the speed of resonance *v*(*t*) into a resonance frequency *v*(*f*) using the autospectrum function.

The software enabled to analyze the continuously monitored input signal from the laser vibrometer. The measurement period was set at 31.25 ms. The time signal *v*(*t*) was processed through a fast Fourier transformation (“FFT”) analysis *V*(*f*), resulting in a frequency span ranging between 0 and 12.58 kHz with 400 frequency intervals, resulting in a frequency resolution of 32 Hz. The FFT analysis generates a so-called autospectrum, based on the following formulae:

$$ {S}_v(f)=\sqrt{V(f)\bullet {V}^{\ast }(f)} $$

with *V* ^∗^(*f*) being the complex conjugate of *V*(*f*).

**Fig. 3:**
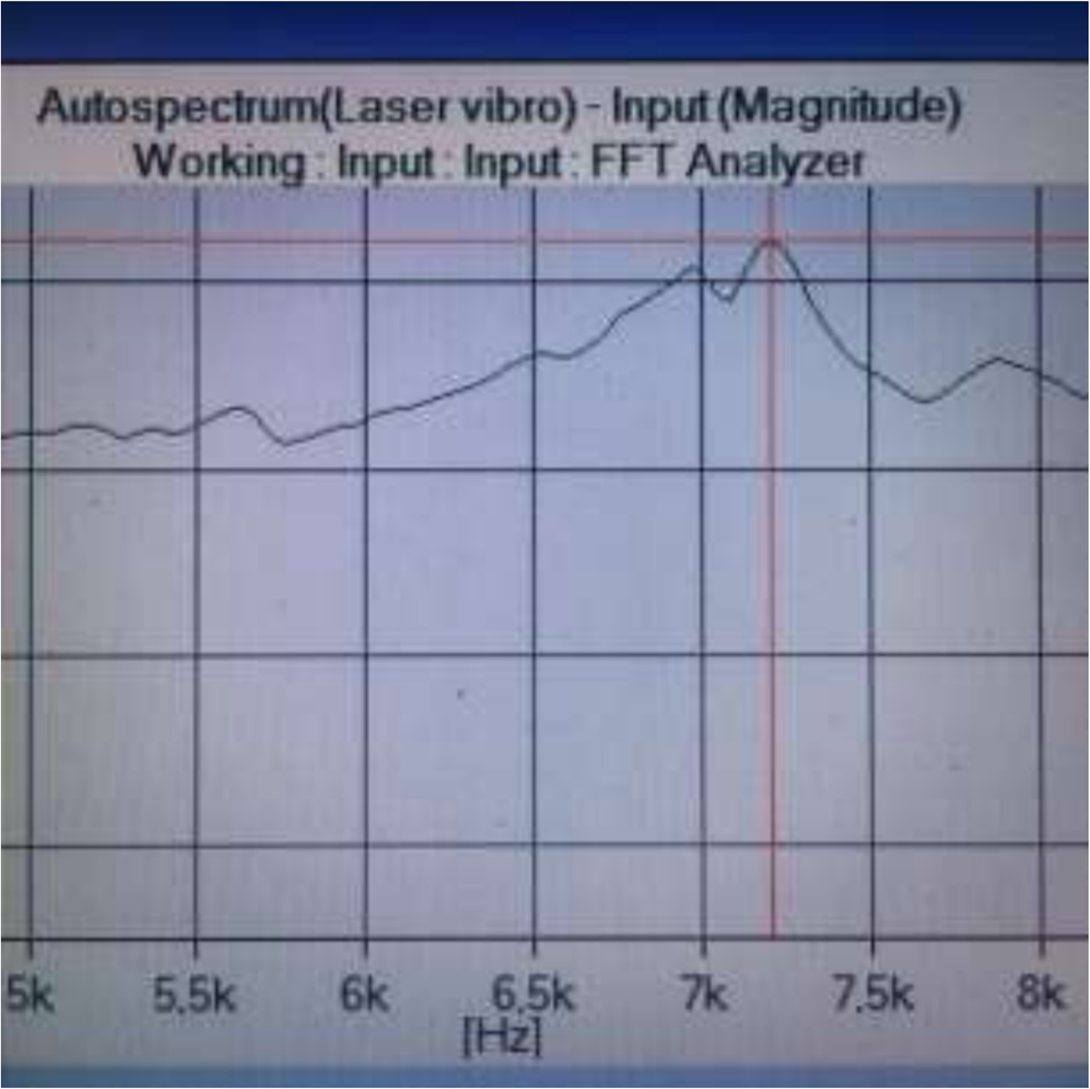
Example of a typical autospectrum pointing to a 1 maximum RF based on 1000 measurements in case of a Straumann test implant

The final generated autospectrum pointed the maximum resonance frequency value based on an average of 1000 measurements per detection session (Fig. 3). This recorded maximum resonance vibration frequency was noted in the datafile. The measurement was done in fivefold, and a mean value of all five measurements was computed, serving as the value to be input in the Osstell algorithm. Secondly, the “direct” ISQ value generated by the Osstell device was also noted in the datafile. After completion of measurements for each out of the five implants in each resin blocks, measurements were repeated in fourfold. In total, five measurements were made for each test implant.

In total, for each given implant type with a given diameter/length configuration, 25 measurements for indirect and 5 measurements for direct ISQ computing were performed.

### Statistics

The SPSS statistical software package 22.0 (IBM SPSS, Chicago, USA) was used. A Shapiro-Wilk test was used to verify distribution normality for both direct and indirect determined ISQ values. The paired sample *t* test and the Wilcoxon signed rank test were used to evaluate the match between direct and indirect ISQ values. The Pearson product-moment correlation coefficient was used to assess the strength of the linear relationship between direct and indirect determined ISQ values. A 0.05 *p* value was used as type I error.

## Results

Mean values (± SD) of recorded maximum RF values, calculated indirect ISQ values, and direct recorded ISQ values for Ankylos (A) and Straumann (S) test implants are shown in Table 2.

**Table 2:** Mean values (± SD) of recorded maximum RF values, calculated indirect ISQ values, and direct recorded ISQ values for Ankylos (A) and Straumann (S) test implants

Batch #	Implant system	Implant length (mm)	Implant diameter (mm)	Mean measured resonance freq (kHz)	SD measured resonance freq (kHz)	Mean indirect ISQ	SD indirect ISQ	Mean direct ISQ	SD direct ISQ
26	A	9.5	4.5	8607.60	0.894	89.19	0.006	90	0
27	A	9.5	4.5	8000.00	0	85.02	0	85	0
28	A	9.5	4.5	8032.00	0	85.24	0	85	0
29	A	9.5	4.5	8256.00	0	86.77	0	85.8	1.0954
30	A	9.5	4.5	8256.00	0	86.77	0	87	0
40	S	12	3.3	5180.00	3.346	65.63	0.025	65	0
41	S	12	3.3	5180.00	2.828	65.62	0.0212	65	0
42	S	12	3.3	5180.00	2.828	65.62	0.0212	65	0
43	S	12	3.3	5180.00	2.828	65.62	0.0212	65	0
44	S	12	3.3	5038.40	2.190	64.54	0.0168	64	0
ts1	S	4.1	10	7257.60	26.773	79.95	0.1814	80	0
ts2	S	4.1	10	7251.20	17.5271	79.90	0.1187	80	0
ts3	S	4.1	10	7225.60	14.3108	79.93	0.0968	80	0
ts4	S	4.1	10	7206.40	14.3108	79.60	0.0968	80	0
ts5	S	4.1	10	7232.00	0	79.77	0	80	0
55	S	4.8	8	7070.80	17.922	78.68	0.1209	75	0
56	S	4.8	8	7100.00	30.4302	78.88	0.2055	79	0
57	S	4.8	8	6857.60	21.4196	77.24	0.1441	77	0
58	S	4.8	8	7375.20	41.8473	80.7448	0.2842	77	0
59	S	4.8	8	7115.20	31.5150	78.9835	0.2129	78	0

### Normality of indirect (calculated) versus direct generated ISQ values

Using the Shapiro-Wilk test for indirect ISQ (*p* = 0.05) and direct ISQ (*p* = 0.02), we can conclude that both indirect and direct ISQ measures are not drawn from a normal distribution (data not shown). Both variables show a negative skewness and kurtosis (skewness indirect ISQ = − 6.22; skewness direct ISQ = − 0.491; kurtosis indirect ISQ = − 0.786; kurtosis direct ISQ = − 0.850).

### Match between indirect (calculated) versus direct generated ISQ values

**Fig. 4:**
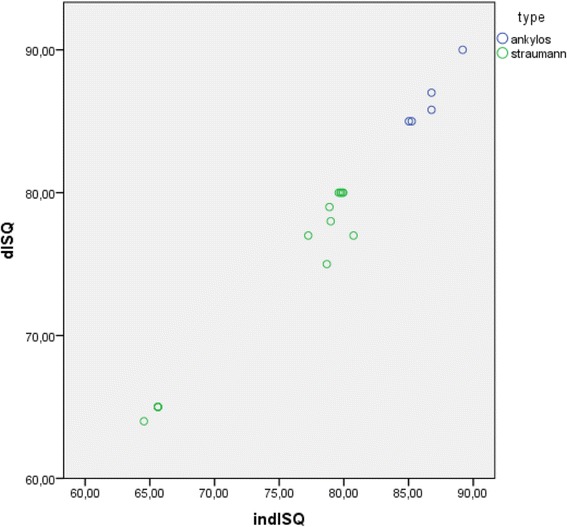
Scatterplot depicting indirect calculated and direct measured ISQ values of the tested implants

The mean indirect ISQ value is on average 0.535 IDSQ units higher than the direct ISQ value although this is not significantly different from 0 (paired *t* test *t* = 2.018, df = 19, *p* = 0.058). This is confirmed by the Wilcoxon signed rank test (*Z* = 1.867, *p* = 0.062). Figure 4 graphically represents the match between both outcome variables for all test implants. Differences are noted between Ankylos and Straumann implants and furthermore between the different length-diameter clusters of the Straumann implants.

### Correlation between indirect (calculated) versus indirect generated ISQ values

The Pearson product-moment correlation between indirect and direct ISQ values equals 0.990 with *p* = 0.000 indicating a significantly high linear relationship between both measures.

## Discussion

The focus of this in vitro study was to develop a laboratory method, intended for future research of aspects of implant-Smartpeg complex stiffness and its possible influence on the overall RFA-based implant stability determination. In the past, other laboratory methodologies have been engineered to investigate implant deflection and/or lateral displacement by means of transducers. A setup using a motorized load transducer enabling to impact imbedded implant through a customized mounted abutment in combination with a micrometer gauge is described [18]. Furthermore, induction of resonant vibration on imbedded implants by an impulse-forced hammer, detection of the vibration signal by an acoustic microphone, and subsequent signal processing by fast Fourier transformation are described [19].

By means of the above-described laboratory setup, quantitative measurement of maximum resonance frequency was performed after Smartpeg stimulation with subsequent indirect calculation of ISQ values using the Osstell algorithm. These indirect computed ISQ values were compared to directly determined ISQ values through the Osstell IDx device. The comparison of the indirect and direct ISQ datasets enabled to evaluate the correctness of the laboratory procedure by using the algorithm proposed by Osstell. Since the signal processing software provided a maximum resonance frequency based on 1000 recorded excitation measurements for each single analysis with a frequency resolution of 32 Hz, a measurement technique with high power could be obtained. The calculated ISQ values matched well with the directly generated ISQ values recorded by the IDx device. The difference between indirect and direct ISQ was rejectable from a clinical point of view.

## Conclusions

In conclusion, the present study demonstrated that the algorithm applied and provided by Osstell to calculate ISQ values is correct, making the laboratory procedure valuable for future research focused on stiffness aspects of the implant-Smartpeg complex and its possible influence on the overall RFA measurement. Vice versa, the present study demonstrates the correctness of the actual applied algorithm for calculation of ISQ values by Osstell Mentor and Osstell IDx devices. From a clinical perspective, this study adds proof to the finding of ISQ values obtained by original Osstell devices, at least for Straumann tissue level SLA-surfaced implants, that are biased and underestimated. This implies that comparison of implant stability in terms of ISQ for identical implant systems between different studies has to be done with caution and need to be corrected when used for comparisons in systematic reviews.
